# Reducing SAR in 7T brain fMRI by circumventing fat suppression while removing the lipid signal through a parallel acquisition approach

**DOI:** 10.1038/s41598-021-94692-6

**Published:** 2021-07-28

**Authors:** Amir Seginer, Edna Furman-Haran, Ilan Goldberg, Rita Schmidt

**Affiliations:** 1 Siemens Healthcare Ltd, Rosh Ha ayin, Israel; 2 Life Sciences Core Facilities, Weizmann Institute of Science, Rehovot, Israel; 3 Deparment of Neurology, Wolfson Medical Center, Holon, Israel; 4 Neurobiology Department, Weizmann Institute of Science, Rehovot, Israel

**Keywords:** Imaging techniques, Biomedical engineering

## Abstract

Ultra-high-field functional magnetic resonance imaging (fMRI) offers a way to new insights while increasing the spatial and temporal resolution. However, a crucial concern in 7T human MRI is the increase in power deposition, supervised through the specific absorption rate (SAR). The SAR limitation can restrict the brain coverage or the minimal repetition time of fMRI experiments. In the majority of today’s studies fMRI relies on the well-known gradient-echo echo-planar imaging (GRE-EPI) sequence, which offers ultrafast acquisition. Commonly, the GRE-EPI sequence comprises two pulses: fat suppression and excitation. This work provides the means for a significant reduction in the SAR by circumventing the fat-suppression pulse. Without this fat-suppression, however, lipid signal can result in artifacts due to the chemical shift between the lipid and water signals. Our approach exploits a reconstruction similar to the simultaneous-multi-slice method to separate the lipid and water images, thus avoiding undesired lipid artifacts in brain images. The lipid-water separation is based on the known spatial shift of the lipid signal, which can be detected by the multi-channel coils sensitivity profiles. Our study shows robust human imaging, offering greater flexibility to reduce the SAR, shorten the repetition time or increase the volume coverage with substantial benefit for brain functional studies.

## Introduction

Ultra-high field (≥ 7T) magnetic resonance imaging (MRI) offers both an increased signal-to-noise ratio (SNR) and an improved contrast-to-noise ratio (CNR), which can be exploited to increase the spatial and temporal resolution. One of the methods that has harnessed these benefits is functional MRI (fMRI). The majority of fMRI experiments rely on the Blood-Oxygen-Level-Dependent (BOLD) contrast that captures dynamic T_2_^*^ changes^1^. Multiple sequences are used and developed for different fMRI applications, including FLASH^1,2^ and GRE-EPI^3,4^. When more localized T_2_ contribution is of-interest, spin-echo EPI fMRI experiments are also performed, especially at 7T^5^. Nevertheless gradient-echo echo-planar imaging (GRE-EPI) has become the method of choice for fMRI due to its ultrafast acquisition^6^ and T_2_^*^ sensitivity. However, EPI is also noted for its low effective bandwidth in the phase encoding (PE) direction^7^. One of the consequences of this low bandwidth is the introduction of a large lipid-water shift in the image due to the chemical shift between the lipid and water signals, resulting in artifacts in the image. The shift proportionally increases with the strength of the magnetic field (for a given EPI readout train). The lipid artifacts can reduce image quality and fMRI efficiency, particularly since the BOLD effect itself is small compared to the signal and is prone to any undesirable artifacts. An effective technique commonly used to remove these artifacts is to prepend an RF pulse to suppress the lipid signal. Therefore, a common GRE-EPI sequence is comprised of two pulses—fat suppression and excitation. When targeting whole-brain coverage (with a high slice resolution) and a reduced repetition time, fMRI in 7T can reach the limits of the allowed specific absorption rate (SAR). The fat-suppression pulse can double and even triple the total SAR, depending on the scan parameters. The SAR of the scan also depends on the number of simultaneously acquired slices in simultaneous-multi-slice (SMS) method, since the SAR of the multi-band pulse is increased for higher number of bands. Skipping the fat-suppression pulse can allow a further increase of the SMS factor. In addition, increased RF field inhomogeneity in ultra-high field MRI can locally reduce the efficiency of fat suppression.

Several works have sought to improve the fat-suppression techniques by addressing the RF field inhomogeneity and by reducing the SAR. These studies include SAR-optimized pulses and adiabatic pulse implementations^8–11^. A wide range of other efforts have been directed to developing techniques for the removal of the lipid signal, such as inversion recovery pulses^12^, water excitation^13^ and opposite sign gradient in spin-echo implementation^14,15^. Recent studies have explored the use of methods based on fingerprinting^16,17^ and compressed sensing^18,19^ to reconstruct separate images of the lipid and water.

In this study, we examine the potential to significantly reduce the SAR at 7T by circumventing the fat-suppression pulse. This has already been demonstrated for 3D-EPI where a simple non-spatially selective, but water selective, excitation pulse can be used^11,13,20^. However, this is not easily converted to multi-slice 2D-EPI. To complement the removal of the fat-suppression pulse, we utilized a reconstruction based on the parallel acquisition technique to separate the lipid and water images. An EPI implementation without fat suppression can offer fMRI studies greater flexibility to reduce the SAR, shorten the repetition time or increase the volume coverage.

Since the introduction of SENSE^21^ and GRAPPA^22^, parallel acquisition methods that accelerate the scan and shorten the echo time have proven highly efficient. Reconstruction methods are constantly being improved, including implementations such as ESPIRIT^23^ and simultaneous multi-slice for EPI with CAIPIRINHA^24^. Moreover, the efficiency of parallel acquisition is further enhanced by advances in coil technology and increase in the magnetic field^25^. SENSE and GRAPPA based techniques have also been examined in applications other than acceleration. Examples include the correction of ghost artifacts in EPI^26^ and restricted FOV imaging^27,28^. Parallel acquisition has also been shown to improve the reconstruction in chemical shift imaging^29^ and in lipid ghosts elimination^30^. The SENSE method has been successfully applied in hyperpolarized ^13^C metabolic imaging to reconstruct separate metabolite images^31^; based on their chemical shift. In several feasibility studies SENSE has also been demonstrated to separate lipid and water images from EPI acquisitions at 1.5 T and 3 T MRI^32–34^. In addition, a blipped-CAIPI method was specially designed to correct for the chemical shift ghost for a better slice-selection process^35^. In the current study, we further explore lipid water separation for EPI in 7 T MRI. The separation is based on the distinct chemical/spatial shift of the lipid signal that can be detected by the multi-channel coils’ sensitivity profiles. This can be visualized as analogous to CAIPIRINHA’s^36^ shift of the slices in simultaneous-multi-slice (SMS) acquisition. In the lipid-water case, the introduced FOV shift is defined not by added gradient blips but rather by the effective bandwidth in the PE direction and the chemical shift of the lipid peak (3.4 ppm which corresponds to ~ 1000 Hz at 7 T). The large lipid-water signal shift in the EPI images at 7 T actually aids reliable reconstruction. However, note that despite its benefit for lipid-water separation, the low effective PE bandwidth is also responsible for increased image distortions in EPI. Both—the lipid-water shifts and the distortions—are reduced by in-plane parallel acquisition. Thus, there is a tradeoff between maximizing the lipid-water separation and minimizing the image distortions.

In this study, lipid-water separation is examined in combination with parallel acceleration—including both in-plane and SMS. The benefits of the proposed strategy are examined, including the substantial reduction in the SAR. One of the concerns is the potential impact of the lipid-water separation on the accessible acceleration factor as it uses the same coil information resources. To examine this issue, we estimated the geometry factor (g-factor) maps for lipid-water separation compared to SMS acceleration. In addition, human volunteer scanning was performed to examine the reconstruction quality in-vivo and to assess fMRI efficiency. Note that realistic fat suppression pulses typically reduce the SNR of the acquired image, so avoiding the fat suppression pulse has the potential of increasing the SNR^37^ and the temporal SNR (tSNR) accordingly^38^. In this study, we also examined the tSNR and compared it between experiments with and without a fat-suppression pulse.

### Lipid and water images separation: principles and extended parallel imaging formulation

Lipid-water images can be reconstructed separately using the parallel imaging technique if distinct sensitivity profiles exist as was demonstrated in Ref.^34^. The combined lipid-water acquired signal can be described as follows:

1 $$\left[ {\begin{array}{*{20}c} {S_{ch = 1} } \\ {S_{ch = 2} } \\ {\begin{array}{*{20}c} \vdots \\ {S_{{ch = N_{ch} }} } \\ \end{array} } \\ \end{array} } \right] = {\mathcal{F}}\left( {\left[ {\begin{array}{*{20}c} {\begin{array}{*{20}c} {C_{w\,ch = 1} } \\ {C_{w\,ch = 2} } \\ {\begin{array}{*{20}c} \vdots \\ {C_{{w\,ch = N_{ch} }} } \\ \end{array} } \\ \end{array} } & {\begin{array}{*{20}c} {C_{lip\,ch = 1 } } \\ {C_{lip\,ch = 2 } } \\ {\begin{array}{*{20}c} \vdots \\ {C_{{lip\,ch = N_{ch} }} } \\ \end{array} } \\ \end{array} } \\ \end{array} } \right]\left[ {\begin{array}{*{20}c} {\rho_{w} } \\ {\rho_{lip} } \\ \end{array} } \right]} \right)$$

where $S_{ch}$ is the k-space acquired signal per channel (*ch* = *1..*$N_{ch}$), ${\mathcal{F}}$ is the FFT operator, *C*_*lip*_ and *C*_*w*_ are the per-channel sensitivity profiles of the lipid and water signals, respectively, and *ρ*_*lip and*_* ρ*_*w*_*,* are the lipid and water images, respectively . *C*_*lip*_ can be generated by either shifting the *C*_*w*_ by the known lipid-water spatial shift or by acquiring an additional lipid image (e.g., by using a water suppression pulse) and shifting it by the same known lipid-water shift. Note that this formulation is analogous to SMS reconstruction.

The shift of lipid (*d*_*lip-w*_) in EPI is defined as follows:

2 $$\frac{{d_{lip - w} }}{FOV} = \frac{{\Delta F_{lip - w} }}{{\left( {\frac{1}{{t_{esp} }}} \right) \cdot R_{PE} }}$$

where *FOV*— is the FOV along the PE direction, ${\Delta F}_{lip-w}$ is the spectral shift of lipid from water in Hz , *t*_*esp*_ is the echo spacing in seconds and ${R}_{PE}$ is the acceleration factor along the PE direction, if applied. The shift of the lipid in the readout direction in EPI is usually less than a pixel. Using Eq. (2) with FOV of 20 cm and replacing $1/{t}_{esp}$ with readout bandwidth of 150-250 kHz (which is commonly used), the readout shift will be 0.8–1.3 mm. Note, that the lipid layer spectrum includes a range of peaks, arising from a complex molecular structure, however, due to relatively large linewidth in the lipid layer region, the dominant peak centered at 3.4 ppm from the water peak is commonly recognized as the main peak of the lipid layer.

Table 1 shows the spatial shifts for representative sets of EPI scans. A higher in-plane acceleration rate proportionally reduces the shift. Notice that the shifts for commonly used scan parameters are substantial, which, as shown here, can be exploited in favor of a reliable separate reconstruction of lipid and water images. The substantial lipid shifts do not contradict the fact that the image distortions due to the effective PE bandwidth are acceptable.

**Table 1:** Lipid-water shift for representative EPI parameters.

In-plane resolution (mm)	${R}_{PE}$	*t*_*esp*_ (ms)	${d}_{lip-w}$(pixels)	${d}_{lip-w}$(mm)	${d}_{lip-w}$∕FOV ratio
1.6 × 1.6	1	0.57	72	115	0.56
1.6 × 1.6	2	0.57	36	56	0.26
1.4 × 1.4	2	0.68	50	70	0.34
1.2 × 1.2	3	0.74	44	51	0.25

To implement a “fit for all” reconstruction method for all three parallel imaging aspects—(1) in-plane PE acceleration, (2) SMS acceleration, and (3) lipid-water separation—the problem was reformulated in a manner that treats all three aspects as if they were case (1), i.e., in-plane PE acceleration. This is achieved by solving a “full-FOV” image defined as the concatenation of all final images (all the slices of the separate water and lipid images), similarly to previous works without lipid-water separation^39–41^. Notice that this extended formulation, further detailed below, was established for convenience and supports any of the developed and well-established reconstruction methods for parallel imaging. This formulation simplifies the reconstruction procedure, since it utilizes the same engine to solve the inverse problem. The prerequisites for reconstruction are only the input signal and a set of sensitivity maps.

For simplicity, let us first describe the case of the acquisition of *R*_*sms*_ simultaneous slices together with an in-plane PE acceleration. The acquired signal per channel can be described in the following manner:

3 $$S_{ch} = {\mathcal{A}}_{PE} \left( {{\mathcal{F}}\left( {\mathop \sum \limits_{sl = 1}^{Rsms} C_{ch} \rho_{sl} } \right)} \right)$$

where ${\mathcal{F}}$ is the FFT operator, ${\mathcal{A}}_{PE}$ is a PE subsampling operator mimicking the actual acquisition that samples only every *R*_*PE*_ line, and $\mathop \sum \limits_{sl = 1}^{Rsms} C_{ch} \rho_{sl}$ is the sum of the simultaneously acquired slices, where *sl* = *1..R*_*sms*_ is the slice counter. In a matrix form, the dimension of the signal $S_{ch}$ is *(N*_*PE*_* ∕R*_*PE*_*)* x *N*_*RO*_, where *N*_*PE*_ and *N*_*RO*_ are the number of points along the PE and RO directions, respectively.

In the extended formulation proposed here, we first stack the slices in the PE dimension, to form an *(R*_*sms*_*∙N*_*PE*_*)* x *N*_*RO*_ matrix and then the final signal is subsampled by an acceleration factor *(R*_*sms*_*∙R*_*PE*_*)*, represented by operator ${\mathcal{A}}_{sl - PE}$. This is represented by the following equation:

4 $$S_{ch}^{{\left\{ {\frac{{R_{sms} \cdot N_{PE} }}{{R_{sms} \cdot R_{PE} }} \times N_{RO} } \right\}}} = {\mathcal{A}}_{sl - PE} {\mathcal{F}}\left( {\left[ {\begin{array}{*{20}c} {C_{sl1} } \\ {\begin{array}{*{20}c} {C_{sl2} } \\ \vdots \\ \end{array} } \\ {C_{Rsms} } \\ \end{array} } \right]_{ch}^{{\left\{ {(Rsms \cdot N_{PE} ) \times N_{RO} } \right\}}} \cdot *\left[ {\begin{array}{*{20}c} {\rho_{sl = 1} } \\ {\begin{array}{*{20}c} {\rho_{sl = 2} } \\ \vdots \\ \end{array} } \\ {\rho_{sl = Rsms} } \\ \end{array} } \right]^{{\left\{ {(Rsms \cdot N_{PE} ) \times N_{RO} } \right\}}} } \right)$$

The matrix sizes are indicated here in the curly brackets. The .* stands for element-by-element multiplication and *C*_*sl*_ are per-channel sensitivity profiles of the slices.

**Figure 1:**
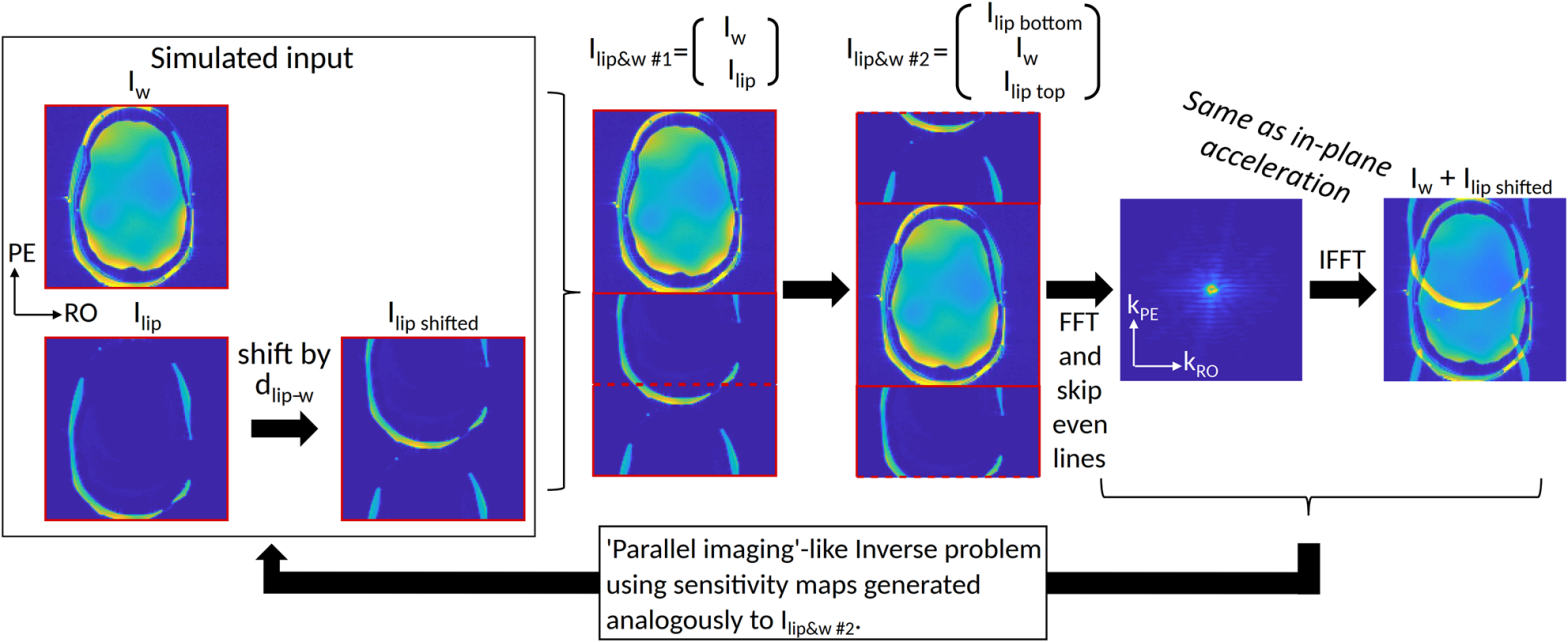
Extended formulation that can combine three parallel imaging aspects (1) in-plane PE acceleration, (2) SMS acceleration and (3) lipid-water separation. The steps show how a resulting EPI image can be described for water and lipid “slices” (I_w_ and I_lip_). The I_w_ and I_lip __shifted_ are concatenated, then shifted together by a half-FOV. Images “I_w_ + I_lip shifted_” (at the far right) and “I_lip__&w__#2_” resemble the case of in-plane acceleration, with “I_w_ + I_lip shifted_” analogous to the acquired image (due to sub-sampling) and “I_lip__&w__#2_”analogous to the full-FOV image in such a case. Following this description, one can solve the inverse problem analogous to in-plane parallel imaging using sensitivity maps. The same description holds also for two slices acquired simultaneously (instead of lipids and water signals). Finally, the formulation can be extended to any number of slices and fat/water images.

Having acquired the signal (left-hand side) and the sensitivity maps, we can solve the inverse problem of Eq. 4 to estimate the slice images (${\rho }_{sl=1,}{\rho }_{sl=2}, .., {\rho }_{sl=Rsl}$), just like in a regular in-plane parallel imaging problem. Note that this holds for an odd number of slices. For even number of slices, the whole stack of slices has to be shifted by a half-FOV, to center a slice at the center of the new effective (stacked) image. This is explained schematically in Fig. 1 and in more detail in the Supporting Information S1. Therefore, for an even number of slices, concatenating the slices will give us:

5 $$S_{ch}^{{\left\{ {\frac{{R_{sms} \cdot N_{PE} }}{{R_{sms} \cdot R_{PE} }} \times N_{RO} } \right\}}} = {\mathcal{A}}_{sl - PE} {\mathcal{F}}\left( {\left[ {\begin{array}{*{20}c} {C_{sl1\ bottom} } \\ {\begin{array}{*{20}c} {C_{sl2} } \\ \vdots \\ \end{array} } \\ {\begin{array}{*{20}c} {C_{Rsms} } \\ {C_{sl1\ top} } \\ \end{array} } \\ \end{array} } \right]_{ch}^{{\left\{ {(Rsms \cdot N_{PE} ) \times N_{RO} } \right\}}} \cdot *\left[ {\begin{array}{*{20}c} {\rho_{sl1\ bottom} } \\ {\begin{array}{*{20}c} {\rho_{sl2} } \\ \vdots \\ \end{array} } \\ {\begin{array}{*{20}c} {\rho_{Rsms} } \\ {\rho_{sl1\ top} } \\ \end{array} } \\ \end{array} } \right]^{{\left\{ {(Rsms \cdot N_{PE} ) \times N_{RO} } \right\}}} } \right)$$

where “bottom” and “top” refer to the bottom and top halves of the image. Notice that in this formulation, the simultaneous acquisition of two slices is analogous to an in-plane PE acceleration by a factor of two. The description above does not include CAIPIRINHA implementation. If CAIPIRINHA is used, the CAIPIRINHA shift is applied separately for each slice and its sensitivity map to match the applied shift during the scan.

Analogously to Eq. 3 for SMS, the lipid-water separation signal can be written as:

6 $$S_{ch} = {\mathcal{A}}_{PE} ({\mathcal{F}}\left( {C_{w,ch} \rho_{w} + C_{lip,ch} \rho_{lip} } \right)$$

and therefore, in analogy to Eq. 4, can also be described as:

7 $$S_{ch}^{{\left\{ {\frac{{2 \cdot N_{PE} }}{{2 \cdot R_{PE} }} \times N_{RO} } \right\}}} = {\mathcal{A}}_{lip,w - PE} {\mathcal{F}}\left( {\left[ {\begin{array}{*{20}c} {C_{lip\ bottom} } \\ {\begin{array}{*{20}c} {C_{w} } \\ {C_{lip\ top} } \\ \end{array} } \\ \end{array} } \right]_{ch}^{{\left\{ {(2 \cdot N_{PE} ) \times N_{RO} } \right\}}} \cdot *\left[ {\begin{array}{*{20}c} {\rho_{lip\ bottom} } \\ {\begin{array}{*{20}c} {\rho_{w} } \\ {\rho_{lip\ top} } \\ \end{array} } \\ \end{array} } \right]^{{\left\{ {(2 \cdot N_{PE} ) \times N_{RO} } \right\}}} } \right)$$

where ${\mathcal{A}}_{lip,w - PE}$ is the subsampling operator that mimics the actual acquisition, sampling both the lipid and the water images and, therefore, subsamples every 2*∙R*_*PE*_ lines (2 for lipid and water). $C_{w,ch} \rho_{w} + C_{lip,ch} \rho_{lip}$ is the sum of the combined lipid and water signal.

Finally, the lipid-water and slices datasets can be jointly concatenated to give a total effective “acceleration” factor of *R*_*tot*_ = *2∙R*_*sms*_*∙R*_*PE*_ (for a spatial acquisition acceleration factor *R*_*acc*_ = *R*_*sms*_*∙R*_*PE*_). For example, the case of *R*_*sms*_ = 2 with lipid-water separation will be analogous to acceleration by a factor of four, *R*_*tot*_ = 2∙2∙1 (*R*_*acc*_ = 2).

With this formulation in hand, the solution can be based on any parallel imaging reconstruction method, including the well-known SENSE, GRAPPA, ESPIRIT, as well as any other inverse problem solution. In this study, a custom-written MATLAB code was used to arrange the input k-space and the sensitivity profiles. The final reconstruction was performed utilizing the BART^42^ software, using the “pics” command with L1 norm.

## Results

**Figure 2:**
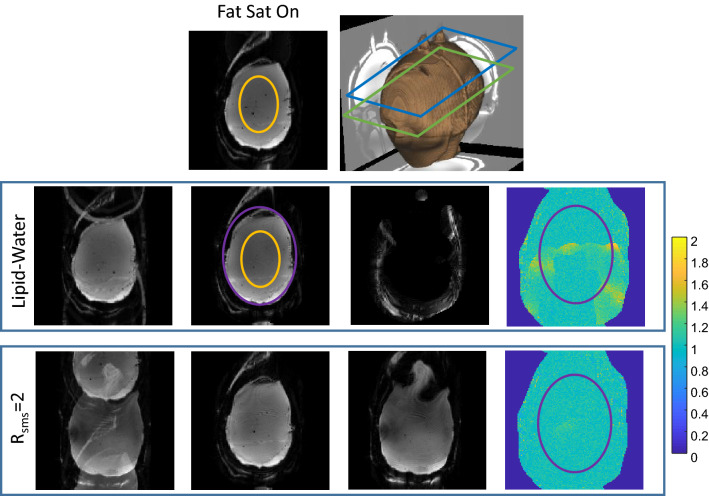
Phantom scanning—comparison of Lipid-Water and SMS reconstruction. Top row—Image acquired with fat suppression and a 3D rendering showing the phantom shape, the main slice location (green frame) and a second slice (blue frame). Second row—Images acquired without fat suppression. From left to right—image with standard reconstruction (with overlapping lipid signal), reconstruction of separated water and lipid images, and a g-factor map for the water image. The lipid image is placed at the correct location (without a shift) by the reconstruction algorithm. Bottom row—Images acquired with R_sms_ = 2. From left to right—image with standard reconstruction (with overlapping slices), reconstructed two slices images and g-factor map for the first slice. The ROIs shown in the orange overlay were used to estimate the SNR, while the purple ROI shows the “brain” region in the images. The g-factor color-map range is 0–2.

Phantom experiments were conducted to examine the reconstruction quality. A 3D-printed head phantom that included lipid and brain compartments was used. Figure 2 compares the lipid-water separation with R_sms_ = 2. The same slice is reconstructed in both cases and the g-factor maps for this slice are compared. The average and standard deviation of the g-factor are 1.06 ± 0.12 for lipid-water separation and 1.01 ± 0.08 for R_sms_ = 2. The maximum g-factors inside the phantom are 1.6 and 1.4 for lipid-water separation and R_sms_ = 2, respectively. The low g-factor for R_sms_ = 2 is due to the CAIPRINHA implementation. Unlike the SMS case, the lipid-water g-factor map shows a localized increase in a narrow range, that of the shifted lipid layer region. The SNR of the image without fat suppression was estimated to be × 1.3 higher than with fat suppression.

**Figure 3:**
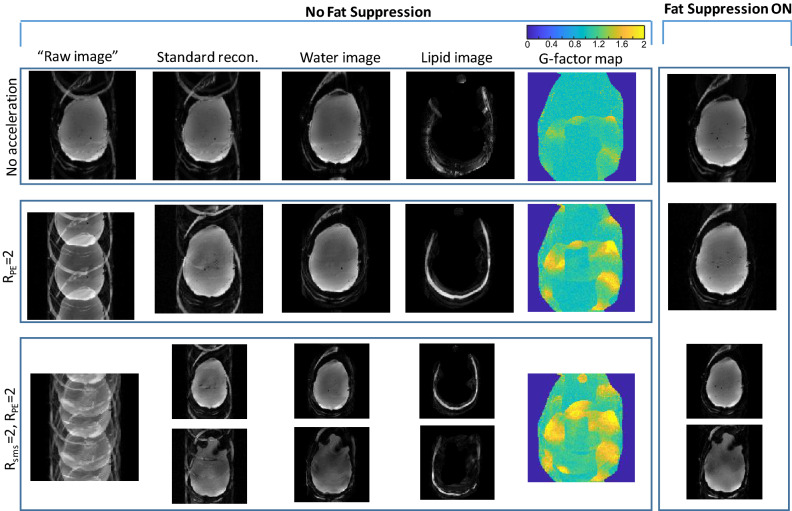
Phantom scanning—combining in-plane acceleration, SMS and lipid-water separation. From top to bottom—*R*_*tot*_ = 2 no acceleration (Lipid/Water separation), *R*_*tot*_ = 4 (Lipid/Water, *R*_*PE*_ = 2), *R*_*tot*_ = 8 (Lipid/Water, *R*_*PE*_ = 2, R_sms_ = 2). From left to right—“raw image” (FFT applied directly to the acquired image), “standard recon.” (Siemens product reconstruction), images reconstructed with the extended formulation—separate water and lipid images, g-factor maps for the common slice, and the image acquired with fat suppression. The g-factor color-map range is 0–2.

Figure 3 shows a further comparison of combined acceleration factors with lipid-water separation. It includes well-reconstructed images with a total acceleration of *R*_*tot*_ = 2 (lipid-water separation only, *R*_*acc*_ = 1), *R*_*tot*_ = 4 (lipid-water separation and *R*_*PE*_ = 2, *R*_*acc*_ = 2) and *R*_*tot*_ = 8(lipid-water separation, *R*_*PE*_ = 2 and R_sms_ = 2, *R*_*acc*_ = 4). The g-factor values increase to 1.18 ± 0.26 for *R*_*tot*_ = 4 and to 1.31 ± 0.28 for *R*_*tot*_ = 8.

Figure S1 in the Supporting Information shows an analysis of the added relative signal of the lipid artifact versus the flip angle of the Fat Suppression pulse, as well as energy fraction the Fat Suppression pulse contributes. Flip angles in the range of 40–180° for the fat suppression pulse were examined in conjunction with an R_sms_ = 2, 90° flip angle excitation pulse (as a representing example). A lipid signal close to zero was measured at a fat suppression flip angle of 125°, compared to a default flip angle of 110° used in this sequence, which contributes ~ 66% of the total energy of the product GRE-EPI. The strategy of reducing the flip angle of the fat suppression can be used to reduce global SAR^37^. For example, a choice of 80° will introduce only ~ 7% lipid artifact. This same flip angle (80° for the fat suppression pulse) was also mentioned as the fat suppression flip angle chosen in Ref.^43^. Note, that the lipid artifact intensity can vary depending on additional factors—such as B_0_ distribution, specific sensitivity maps, TE and TR parameters—introducing negligible artifacts in some cases and strong in others. Nevertheless, an 80° will still have an effect on the total SAR, contributing 59% of the total energy of the pulse-sequence and reducing SAR by 34%. Further optimization can be performed by both reducing the flip angle of the fat suppression pulse and using the fat separation formalism, thus balancing artifact amplitude and SAR.

**Figure 4:**
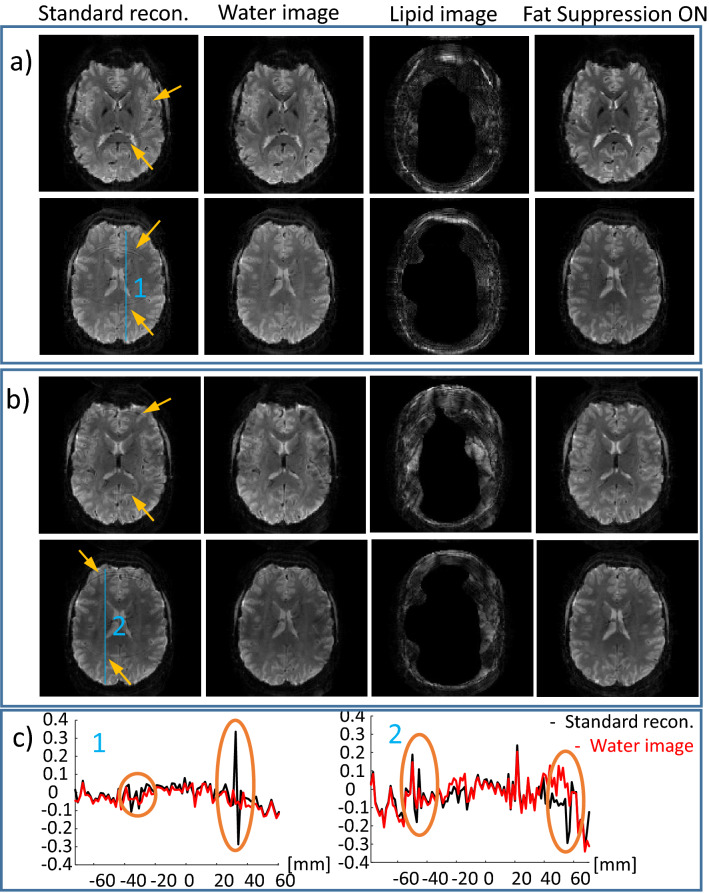
Human imaging – combined in-plane acceleration and lipid-water reconstruction. Two representative slices are shown for *R*_*PE*_ = 2 (**a**) and *R*_*PE*_ = 3 (**b**). From left to right–standard recon. (Siemens product reconstruction), water image, and lipid image from scan without fat suppression, and the image acquired with fat suppression. (**c**) Relative signal change ($\frac{\left({I}_{standard}-{I}_{Fat Sup. On}\right)}{{I}_{Fat Sup. On}}$ and $\frac{\left({I}_{water}-{I}_{Fat Sup. On}\right)}{{I}_{Fat Sup. On}}$) along the blue lines (1) and (2) shown in (**a**) and (**b**), respectively. Yellow arrows point to the artifact due to the lipid signal in the standard reconstruction.

**Figure 5:**
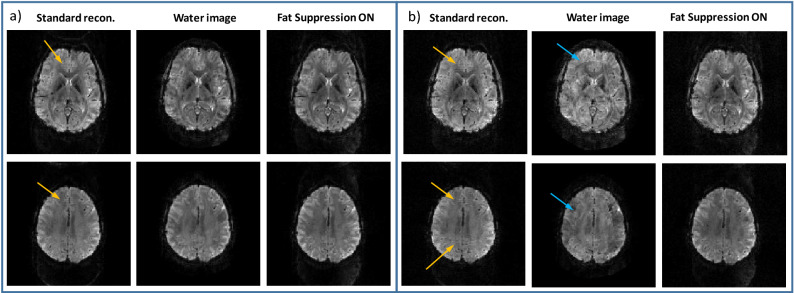
Human imaging—combined in-plane acceleration, SMS and lipid-water reconstruction. Two representative slices are shown for (**a**) *R*_*PE*_ = 2 and R_sms_ = 3 and (**b**) *R*_*PE*_ = 2 and R_sms_ = 4. From left to right—standard recon. (Siemens product reconstruction), water image, and lipid image from scan without fat suppression, and the image acquired with fat suppression. Yellow arrows point to the artifact due to the lipid signal in the standard reconstruction. Blue arrows point to artifacts that occur due to high acceleration factor—R_tot._

Human volunteers were scanned to examine the lipid-water separation in-vivo and to verify the fMRI efficiency. Figures 4 and 5 demonstrate human imaging of two representative slices out of 30 well-reconstructed slices for scans with and without fat-suppression. Figure 4 shows two scans with in-plane resolution of (a) 1.6 mm and (b) 1.4 mm. As increasing the resolution commonly requires higher in-plane acceleration, we examined *R*_*PE*_ = 2 for the first case (1.6 mm) and *R*_*PE*_ = 3 for the second case (1.4 mm). The lipid signal artifact can be observed in the standard reconstruction without fat suppression (see yellow arrows). Examples of a relative signal profile (relative to the fat-suppression case) along two lines are also shown (denoted “1” and “2”). The lipid artifact reaches 35% along line “1”and 30% along line “2” and is practically removed after lipid separation. As can be seen, the separated lipid images include an internal mask (as well as an external one). This masking comes from the threshold-based mask built into the lipid sensitivity maps (generated by the BART software from a lipids only image, see Methods section). Such masking is reasonable due to the precranial localization of the lipids. Figure S3 compares the effect of the masked and non-masked lipids sensitivity maps on the reconstruction (when the non-masked map is generated based on the water image). Examining the water images, no significant differences were observed due to the different choice of the lipids’ sensitivity map. Note that for applications where the lipid image is of interest, further optimization of the sensitivity maps is required as the lipid signal is low and therefore any water residuals in the lipid image will have higher impact on the final lipids image. This optimization is outside the scope of the current work as the focus of this work is on the strong water signal (within the brain).

An additional experiment combining both in-plane and slice acceleration—*R*_*PE*_ = 2 with either R_sms_ = 3 or R_sms_ = 4—is summarized in Fig. 5. The lipid signal artifact, in these cases, can be observed in the standard reconstruction (see yellow arrows). Water images with R_sms_ = 3 (R_tot_ = 12, *R*_*acc*_ = 6) are well-reconstructed, while R_sms_ = 4 (R_tot_ = 16, *R*_*acc*_ = 8) introduce some artifacts (see blue arrows).

In addition, scan parameters for fMRI experiments were examined to identify SAR restrictive cases. Table 2 shows a comparison of SAR levels between fMRI experiments with and without fat suppression. At 7 T, fMRI experiments commonly require in-plane acceleration of factor 2 or 3 to achieve optimal TE (in the range of 20–35 ms, depending on the region of interest). We examined parameters for scans with resolution and slice coverage of interest for fMRI, targeting TE in the above range and with TR in the range of 1.5–2 s, a typically desired temporal resolution. Although SAR is reduced when increasing the SMS factor, in practice, combining in-plane acceleration with higher SMS factors introduces artifacts and reduces SNR—depending on the specific receive coil—and therefore SMS factors of 2 or 3 are of interest. Table 2 shows examples with SMS of factors 2 and 3, which are SAR constrained when fat suppression is applied, but are significantly relaxed if fat suppression is avoided. Cases 1–3 are based on the Siemens product EPI sequence and cases 4–5 on the multi-band GRE-EPI sequence from the University of Minnesota Center for Magnetic Resonance Research (CMRR)^40,44^. The CMRR multi-band GRE-EPI sequence is in wide use in the community, as it offers several complimentary features to the product sequence. Since the CMRR sequence is another GRE-EPI implementation, in addition to that provided by the vendor, with different excitation and fat suppression pulses, it provides another, different, example of the SAR contribution from a Fat Suppression pulse.

**Table 2:** Examples of scan parameters with restrictive SAR.

Sequence	Case	N_sl_	R_PE_	R_sms_	TE (ms)	TR (ms)	SAR (%) Fat. Sup. ON	SAR (%) Fat. Sup. OFF
Ep2d_bold (Siemens product) 1.6 mm iso, flip angle = 90	1	64	3	2	20 (partial Fourier = 7/8)	1500	101	34
	2	60	3	2	21	1500	97	33
	3	76	3	2	21	1500	*	35
cmrr_mbep2d_bold 1.4 mm iso, RF excitation pulse duration = 4 ms, flip angle 90	4	78	3	2	22.6	2030	141	80
	5	78	3	3	22.6	2000	101	59

*This case could not be acquired with TR shorter than 1860 ms (because of the extra time required for the fat suppression pulses)’.

The reference amplitude for 1 ms 180° hard pulse: 240 V.

A set of simulations was also performed to examine the effect of different parameters on the lipid artifact. The simulations were based on brain lipid and brain water gradient-echo images. The lipid image was first shifted to match the EPI shift, similarly to the shift performed in Fig. 2. Then the images were combined and reconstructed using the sensitivity maps to simulate an image without Fat Suppression as well as a water image after the fat separation formalism applied. The deviation of the signal at the lipid artifact was calculated. The T_2_^*^ of the lipid and the gray matter was estimated from a multi-echo GRE scan. The resulting T_2_^*^ was 12.5 ± 2.5 ms and 24.8 ± 1.4 ms in the brain lipid layer and gray matter, respectively. The lipid signal in the simulation was scaled to correspond with the T_2_^*^ and the TE of the EPI acquisition. The results show that without fat suppression the artifact can result in a 23% error. The water image reconstructed by the fat separation formalism results in less than 1.7% root-mean-square-error (rmse). The simulation also examined small movement that can occur during fMRI experiment (by shifting the sensitivity maps by one pixel), it increased the lipid error without Fat Suppression to 32% and the rmse of the water image (after fat separation) to 3.3%.

**Figure 6:**
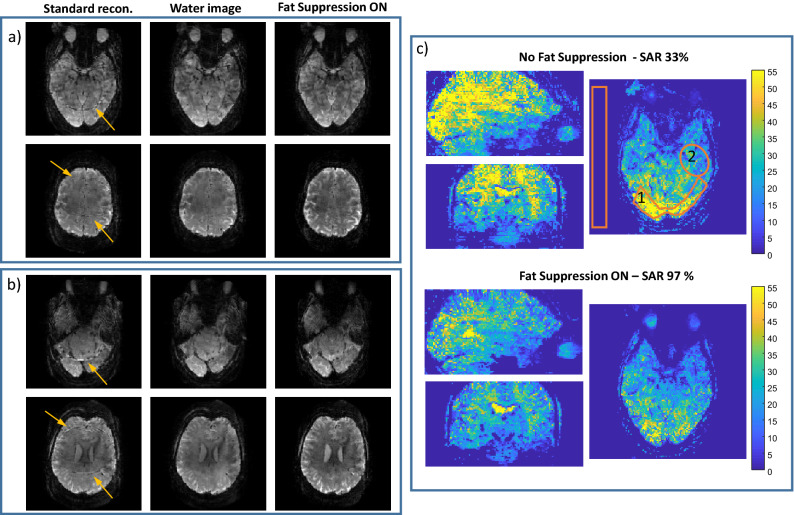
Human imaging—resting-state fMRI scan. Reconstruction of two pairs (**a** and **b**) of simultaneously acquired slices; comparing, left to right, standard reconstruction, a water image after lipid separation and an image acquired with fat suppression. (**c**) tSNR comparison in three orthogonal planes. Yellow arrows in (**a**) and (**b**) point to the artifact due to the lipid signal in the standard reconstruction. The orange overlays in (**c**) show regions (1) and (2) for the SNR and tSNR estimation. The SAR was 33% (without fat-suppression) and 97% (with fat-suppression) where the reference amplitude for 1 ms 180° hard pulse was 240 V.

The human scans also included a five minute-long (200 repetitions) resting state fMRI, both with and without fat suppression, to estimate the SNR and tSNR for each case (summarized in Fig. 6). In this test, a combination of *R*_*PE*_ = 3 and R_sms_ = 2 was applied to achieve a spatial and temporal resolution representative of 7 T- repetition time of 1.5 s and an isotropic resolution of 1.7 mm. This acceleration results in *R*_*tot*_ = 12 when lipid-water separation is included in the reconstruction. A pair of simultaneously acquired slices are shown without and with lipid-water separation compared to applying fat-suppression. In (c) tSNR maps without and with fat-suppression are compared. The SNR ratio of R_tot_ = 12 [*R*_*acc*_ = 6 (*R*_*PE*_ = 3, R_sms_ = 2), Lipid-Water] without fat suppression to R_tot_ = 6 [*R*_*acc*_ = 6 (*R*_*PE*_ = 3, R_sms_ = 2)] with fat suppression, were 1.35 and 1.23 in regions 1 and 2, respectively. The tSNR ratio was 1.47 and 1.13 in regions 1 and 2, respectively.

Finally, the human scanning included 3 volunteers with 6 repeated fMRI motor-task and 6 resting-state experiments. The motor-task experiment included 8 right hand and 8 left hand blocks of finger tapping with a total scan time of 5 min. The block durations were randomized, resulting in 17 ± 3.5 s block durations. The experiments were repeated with and without fat-suppression (with fat-separation reconstruction). The results are summarized in the Supporting Information section S4. If we define contrast to noise ratio (CNR) as ΔS/StdErr., it was increased by × 1.28 ± 0.19 (average and standard deviation in the repeated experiments). The tSNR in the Posterior Cingulate Cortex (PCC) area increased on average by 1.27 ± 0.29.

## Discussion

Pushing the limits and moving to 7 T MRI allows an increase of the spatial resolution as well as to shorten the repetition time. However, scanning at 7 T also increases the SAR, making it a limiting factor, including in fMRI^45^. Methods to reduce the SAR are, therefore, in high demand. In this study, we demonstrate a method that allows the avoidance of fat suppression in EPI, a method with which the SAR of a GRE-EPI can be reduced by a factor of two or even three. This implementation allows better flexibility in the design of the scan protocol: reducing the SAR, shortening the repetition time or increasing the slice coverage. The method proposed here can be used to optimize the spatial coverage, increasing the number of slices and/or reducing the slice thickness without exceeding the SAR limit. As fMRI experiments commonly require long repeating scans with a total duration above 30 min, they can therefore benefit tremendously from reducing the total SAR of the experiment (reducing potential accumulated heating of the subject). In addition, Table 2 shows examples in which circumventing fat suppression will achieve a larger number of slices or shorter TR, with resolution and slice coverage that are of interest in fMRI. Cases 1–2 show SAR close to 100% with fat suppression, which is reduced by a factor of 3 when fat suppression is avoided. Case 3 shows 30% better slice coverage (76 vs 60 slices) for a TR of 1500 ms. Case 3 also demonstrates a 25% shorter TR compared to case 4 (with 78 slices instead of 76). This while case 4 as well as case 5 are examples of scans that can only be acquired without fat suppression. Note that SAR reduction depends on the pulses implemented in the sequence.

By skipping the fat-suppression pulse a lipid artifact is introduced. It may be negligible in some cases, however, it may also introduce significant errors in both the anatomical image and in the fMRI analysis. The lipid signal can corrupt the anatomical image, as shown in all figures. Specifically, we show a simulation study (Fig. S4) in which without Fat Suppression the lipid artifact reaches up to 23% of the water signal, depending on the specific phase contributions. If the fMRI region of interest does not coincide with the lipid artifact, the fMRI analysis will not suffer. However, if it does, the estimation of the signal change due to the BOLD effect will be under- or over-estimated. For example, in case of 23% lipid artifact and 10% BOLD signal change, the signal change that we will measure will be 8%. Since in fMRI experiments we are looking for very small changes, such underestimation can be substantial. In addition, the lipid artifacts can be further increased due to a small movement during the fMRI repetitions. In Fig. S4e a movement by one pixel is simulated, increasing the lipid artifact from 23 to 32%. Therefore, skipping the fat-suppression pulse requires an alternative method to remove the lipid signal from the generated image. In this study, a reconstruction based on an SMS-like parallel acquisition reconstruction was demonstrated to separate water and lipid images. An extended formulation was outlined in this work to combine in a single formulation lipid-water separation, in-plane acceleration and SMS acceleration.

Lipid-water separation was compared to R_sms_ = 2 in Fig. 2. The average g-factor only slightly increased (5%) for the lipid-water case, however, the standard deviation increased by × 1.5. This rise in the standard deviation is because the main change in the g-factor maps is localized to the region of the shifted lipid. The g-factor in Fig. 3 reaches > 2 in the edges of the lipid artifact region. We believe that such high values are related to the suboptimal sensitivity maps in the lipid layer edges. Further work on improvement of the sensitivity maps can improve the results. The SNR, as with SMS using CAIPIRINHA, is reduced when the g-factor is increased. In addition, the SNR of the acquired image actually improves when the fat-suppression pulse is removed, therefore one can further benefit from using this reconstruction method. Both phantom and human imaging showed an SNR improvement of ~ 30%. tSNR was estimated in a resting-state fMRI scenario and demonstrated a 12–50% increase upon removal of the fat-suppression pulse. An fMRI motor-task study showed improved statistics, increasing the number of voxels with t-test ≥ 2.4 (p-value of 99% confidence) by > 1.3.

Total acceleration factors (*R*_*tot*_) of 2–16 (*R*_*acc*_ of 1–8), including a factor of 2 for the lipid-water separation, were demonstrated in phantom and in human imaging. Phantom imaging is commonly prone to larger artifacts due to its uniformity, yet we were able to reconstruct high-quality lipid and water images. In human imaging, the lipid artefact—when using standard reconstruction without a fat-suppression pulse—can result in a local intensity deviation higher than 30%. The reconstruction method demonstrated here removed the lipid signal. The reconstructed images with either lipid separation or fat suppression showed similar results for the R_PE_ = 2, R_sms_ = 3 case, which represents R_acc_ = 6, i.e., R_tot_ = 12 (Figs. 5 and 6). However, combining R_PE_ = 2 and R_sms_ = 4 with lipid/water separation—resulting in R_tot_ of 16—showed artifacts (Fig. 5), indicating the potential limits of this method. These potential limits are the total acceleration limits which should not depend much on whether the coils information is used for spatial acceleration only or for fat–water separation as well. The total achievable acceleration factor of ~ 12 is supported by other works. For example reference^45^ compares different acceleration factors examined in the Human Connectome Project, with maximum acceleration factor of 10. Thus, currently, the lipid separation method limits the spatial acceleration factor to ~ 6. Extensive work is in progress to further improve the parallel acquisition reconstruction with additional regularization methods and machine-learning techniques^46–48^. These methods could be incorporated in the extended formalism discussed here to further improve the achievable factors.

fMRI scans can benefit extensively from avoiding the fat suppression, which will reduce the SAR in 7 T human imaging. In addition, the lower SAR can further be used to shorten the repetition time or to include more slices. The current study demonstrates a reliable reconstruction of separate lipid and water images using the parallel imaging technique. However, it also introduces an additional factor that eventually competes for the limited resources available to accelerate the acquisition, defined by the sensitivity maps of the multi-channel coil. It can be seen in Fig. 3 that the g-factor values are increased when the total acceleration factor is higher. Further research is required to optimize the FOV shift chosen for CAIPIRINHA when combined with lipid-water separation. In addition, one of the methods to reduce the parallel acquisition acceleration factor is combining the acquisition with the Compressed Sensing^49^ method. Of further interest is spin-echo EPI, since in this sequence the lipid artifact may be stronger due to it being refocused (T_2_ weighted instead of the lipids’ shorter T_2_^*^ weighting) as well as the sequence being more SAR limited. Skipping the fat suppression in this case will have a much lower contribution in reducing the SAR, but eliminating the lipid artifact can still be of high interest for the image quality in such cases. Several studies examine methods to circumvent the need for fat suppression pulse in spin-echo EPI, including longer refocusing pulse^8^ and gradient reversal technique^50^. The method in this study can be also extended to SE-EPI.

## Methods

### Phantom experiments

All scans in this study were performed on a 7 T MRI system (MAGNETOM Terra, Siemens Healthcare, Erlangen, Germany) using a commercial 1Tx/32Rx head coil (Nova Medical, Wilmington, MA). A 3D-printed head-shaped phantom was used for imaging (see Ref.^51^ for details, based on^52^). The inner compartment and the bottom outer compartment were filled with an agar mixture to mimic brain properties. The walls of the upper outer compartment were covered by a thin agar layer to mimic the muscle and skin layers while the space between the two layers was filled with peanut oil to mimic the precranial lipid layer. This phantom was designed to provide an RF field and B_0_ distribution similar to *in-vivo*, which is essential for 7 T MRI tests. The agar mixture comprised 2.5% agar, 5.5 g/L NaCl and 0.1 mM GdDTPA .

Scans with the following parameters were performed to examine the reconstruction quality and to compare the images with fat suppression to those without it: FOV = 220 × 220 mm^2^, resolution = 1.7 × 1.7 mm^2^ (130 × 130 pixels), slice thickness = 2 mm, TR = 2000 ms and minimal TE for each acceleration factor. Acceleration factors *R*_*PE*_ = 2 and *R*_*sms*_ = 2 were examined. Two sets of gradient-echo (GRE) scans were collected to serve as input for the sensitivity maps, one acquiring both fat and water (without fat suppression) and one acquiring fat only, using water suppression. The first (fat and water) was used to generate the "water" sensitivity map, while the lipids sensitivity map was generated from the fat only image. Due to the lipids’ localization their resulting sensitivity map automatically and conveniently included a spatial mask (via an internal thresholding in the BART command we used), although not a tight one and at the cost of an additional scan. The sensitivity maps were generated by the BART command (in MATLAB) bart(‘ecalib –m1 –r35, < kspace > ’). Here r35 defines a radius of 35 k-space samples from the origin, outside of which samples are discarded and so smoothing the map. The m1 option generates one set of sensitivity maps (per channel). Finally, < kspace > stands for the variable name holding the k-space data of either the water + fat or fat-only scan. The common GRE scan parameters were FOV = 220 × 220 mm^2^, in-plane resolution = 1.7 × 1.7 mm^2^, slice thickness = 2 mm, TE = 3 ms. In the fat + water GRE, the scan specific parameters were: TR = 321 ms and total scan duration = 0:42 min. In the fat only GRE, the scan specific parameters were: TR = 1790 ms, scan duration = 3:52 min.

To estimate the g-factor, based on the pseudo-replica method^53^, noise was randomly generated and added to the input signal 300 times. The g-factor was then estimated as the standard deviation per-pixel of the reconstructed images. In addition, to estimate SNR the scan was repeated 50 times and for each scan the signal was averaged over a central region (orange overlay in Fig. 2). The SNR was then estimated from these 50 values by dividing their average by their standard deviation.

### Human scanning

Four human volunteers were scanned to examine the lipid-water separation in-vivo and to verify the fMRI efficiency. All methods were carried out in accordance with the Weizmann Institute of science guidelines and regulations. All scans were performed according to procedures approved by the Internal Review Board of the Wolfson Medical Center (Holon, Israel) after obtaining informed suitable written consents.

The common scan parameters in Fig. 4 were: FOV = 208 × 208 mm^2^, slice thickness = 2 mm, TR = 2000 ms, 30 slices, TE = 22 ms and 18 ms in sets (a) and (b), respectively. Two GRE scans, one without fat suppression and one with water suppression were collected to serve as the input for generating the sensitivity maps. The common GRE scan parameters were FOV = 208 × 208 mm^2^, resolution = 1.6 mm isotropic, TE = 3 ms. The fat + water GRE scan specific parameters were: TR = 273 ms, total scan duration = 0:35 min. While in the fat only GRE scan, the scan specific parameters were: TR = 509 ms, scan duration = 1:06 min.

Figure 5 summarizes the experiment combining both in-plane and slice acceleration—R_PE_ = 2 with either R_sms_ = 3 or R_sms_ = 4. The common scan parameters for both sets were: FOV = 220 × 220 mm^2^, 1.7 mm isotropic resolution, TE = 28 ms, 60 slices. The TRs were TR = 1500 ms and 1000 ms for R_sms_ = 3 and R_sms_ = 4, respectively. The same GRE scans as in the previous paragraph were performed to generate the sensitivity maps. In addition, scan parameters for fMRI experiments were examined to identify SAR restrictive cases (see Table 2).

Figure 6 shows images acquired in the resting-state fMRI experiment. The SNR without fat suppression relative to the fat suppressed image was estimated at two representative regions (see orange overlays in Fig. 6c). It was calculated as the average signal within each region divided by the standard deviation in a noise-only region (outside the brain). The tSNR maps were calculated as the average (per-pixel) of the repeated scans divided by their standard deviation (per-pixel). The fMRI scan parameters were: FOV = 220 × 220 mm^2^, resolution = 1.7 × 1.7 mm^2^, slice thickness = 1.7 mm, TR/TE = 1500/22 ms. The SAR level of the fMRI scans was examined with and without fat suppression.

Three additional volunteers were scanned for comparison of scans with Fat Separation and Fat Suppression in a basic motor-task (finger tapping) and resting state scans. The experiments were repeated to include a total of six sets that included scans with and without Fat Suppression, for both the motor-task and the resting state. One of the sets in the motor-task was excluded due to severe movement during the scan. The scan parameters were R_PE_ = 3, R_sms_ = 2, 60 slices, FOV = 220 × 220 mm^2^, resolution = 1.7 × 1.7 mm^2^, slice thickness = 1.7 mm, TR/TE = 1500/22 ms, excitation flip angle 80°.

## Supplementary Information

Supplementary Information.
