# Supplementary material for: Reducing SAR in 7T brain fMRI by circumventing fat suppression while removing the lipid signal through a parallel acquisition approach
Source: Sci Rep. 2021 Jul 28;11:15371. doi: 10.1038/s41598-021-94692-6 (PMC8319205; doi:10.1038/s41598-021-94692-6)
Supplement: Supplementary file 1 — Supplementary Information. [file 41598_2021_94692_MOESM1_ESM.pdf]

Supporting Information

Reducing SAR in 7T brain fMRI by circumventing fat suppression while removing the lipid signal through a parallel acquisition approach

Amir Seginer[1], Edna Furman-Haran[2] , Ilan Goldberg[3] and Rita Schmidt[4*]

S1. Extended parallel imaging formulation – including in-plane acceleration, inter-slice acceleration and lipid/water separation

1.1 Two slices – No CAIPIRINHA: Let us assume we have two images, I_1 and I_2, of two different slices. Each image can be divided into a lower half I_{id} and an upper half I_{iu} (i = 1,2):

I_{1u}	I_{2u}
I_{1d}	I_{2d}

We acquire both images simultaneously, using a multiband excitation. Therefore, the measured signal is equivalent to:

$$S = FFT\{I_1\} + FFT\{I_2\} = FFT\{I_1 + I_2\} = FFT\left\{\boxed{\begin{matrix} I_{1u} \\ I_{1d} \end{matrix}} + \boxed{\begin{matrix} I_{2u} \\ I_{2d} \end{matrix}}\right\} \quad (1)$$

Now, assume we want to treat the problem as a parallel imaging problem, as if our final "full-FOV" image is:

I_{2d}		I_{2d}
I_{1u}	=	I_1
I_{1d}		
I_{2u}		I_{2u}

Note that I_{2d} (bottom half of image I_2) is at the top, while I_{2u} is at the bottom. In this way, if we subsample (by a factor of 2) the FFT of this "full-FOV" image, parts I_{2d} and I_{2u} will wrap around to give the same image as in the SMS case.

To solve this new "parallel imaging" problem we need sensitivity maps of our "channels". The sensitivity maps must match the "full-FOV" image (as in SENSE) and so must be of the form

<table>
<tr><td>C_{2d}^{ch}</td><td rowspan="4">=</td><td>C_{2d}^{ch}</td></tr>
<tr><td>C_{1u}^{ch}</td><td rowspan="2">C_{1}^{ch}</td></tr>
<tr><td>C_{1d}^{ch}</td></tr>
<tr><td>C_{2u}^{ch}</td><td>C_{2u}^{ch}</td></tr>
</table>

where C_{id}^{ch} and C_{iu}^{ch} are the two halves of the sensitivity maps of channel *ch* and slice *i* (*i* = 1,2). Finally, the signal can be described:

$$S = FFT\{I_1 + I_2\} = \mathcal{A}_2\mathcal{F}\left(\begin{bmatrix} C_{2d} \\ C_1 \\ C_{2u} \end{bmatrix}_{ch} .* \begin{bmatrix} I_{2u} \\ I_1 \\ I_{2u} \end{bmatrix}\right) \quad , \tag{2}$$

where $\mathcal{F}$ is an FFT operator and $\mathcal{A}_2$ is a factor 2 subsampling operator that mimics the actual acquired dataset. After solving for the “full-FOV” image, the image can be split into the different slices.

1.2 Three slices – No CAIPRINHA: For three slices we use the same principles as above, but we generate the “full-FOV” image as follows:

<table>
<tr><td>I_1</td><td rowspan="3">or</td><td>I_2</td><td rowspan="3">or …</td></tr>
<tr><td>I_2</td><td>I_1</td></tr>
<tr><td>I_3</td><td>I_3</td></tr>
</table>

so that when sub-sampling by a factor of three the “wrapped” image will be the sum of all the slices. Note that here the images were not split into upper and lower halves, but rather used completely, since they wrap around completely. As a consequence, the effective sensitivity maps will be of the form:

C_1^{ch}		C_2^{ch}	
C_2^{ch}	or	C_1^{ch}	or …
C_3^{ch}		C_3^{ch}	

where C_i^{ch} i is the sensitivity map of channel *ch* at slice *i* (*i* = 1,2,3).

1.3 General case - No CAIPIRINHA: In the general case, we always create a "full-FOV" image that has one slice at its center (otherwise, some linear phase has to be added). In the odd case, that is simple; but in the even case, we always have to split one slice in half, putting one half at the top, the other at the bottom. Here are examples for four- and five-slice cases, with I_1 always at the center, but the order can be arbitrary.

I_{3d}		I_3
I_2		I_2
I_1	and	I_1
I_4		I_5
I_{3d}		I_4

The channel sensitivities are analogous.

1.4 CAIPIRNHA case. When using CAIPIRINHA, the same principles as above apply, but the effect of CAIPIRINHA on each slice must be taken into account: The image and sensitivity map within each slice undergoes a wraparound which gradually varies with the slice.

S2. Efficiency and energy contribution of the Fat Suppression pulse

To estimate the efficiency of the fat suppression pulse, an experiment on a phantom was performed, varying the fat suppression pulse flip angle. Flip angles in the range of 40°-180° for the fat suppression were examined in conjunction with an R_{sms}=2 and 80° excitation flip angle (as a representing example). The experiment was repeated for three repetition times (TR). Other scan parameters were as in the fMRI resting state and task experiments: R_{PE}=3, R_{sms}=2, 60 slices, FOV=220x220 mm^2, resolution = 1.7x1.7 mm^2, slice thickness = 1.7 mm, TE = 22 ms. The results are summarized in Figure S1 and Figure S2 shows all 60 slices without Fat Suppression (for TR=1.5 s). In Figure S1.b the lipid signal contribution relative to the water signal is shown. In order to estimate the water signal in the region of the lipid, the water signal on the line shown in FigS1.a was interpolated to the location of the lipid artifact. A lipid signal close to zero was measured

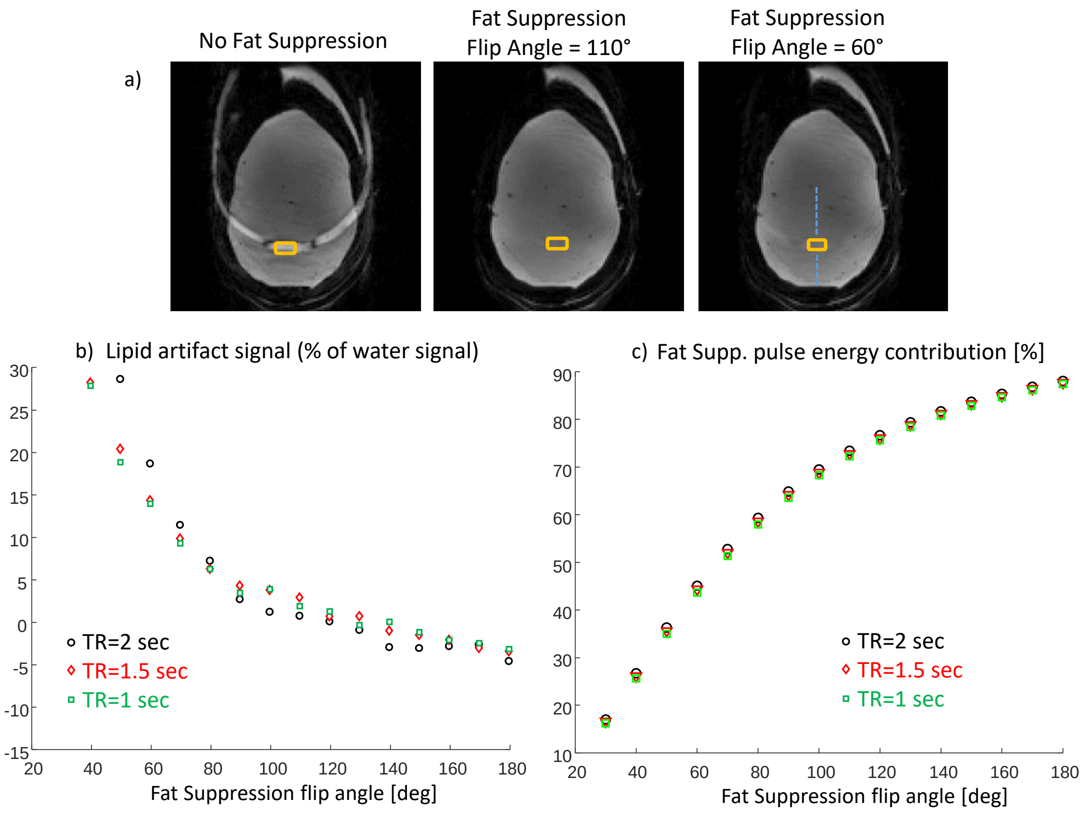

Figure S1: Fat Suppression pulse effect analysis. *a*) Examples of images without Fat Suppression and with Fat Suppression applying different flip angles. b) The lipid artifact magnitude (relative to the water signal) versus the flip angle of the Fat Suppression pulse. c) Fat Suppression pulse energy contribution relative the total energy of the pulse-sequence.

at a fat suppression flip angle of 125°, compared to a default flip angle of 110° used in this sequence. The latter (110°) contributes ~73% of the total energy of the product GRE-EPI. The strategy of reducing the flip angle of the fat suppression can be used to reduce global SAR. For example, a choice of 80° will introduce only ~7% lipid artifact. However, it will still have an effect on the total SAR, contributing 59% of the total energy of the pulse-sequence and reducing SAR by 34%. Further optimization can be performed by reducing the flip angle of the fat suppression pulse together with using the fat separation formalism, thus balancing artifact amplitude and SAR.

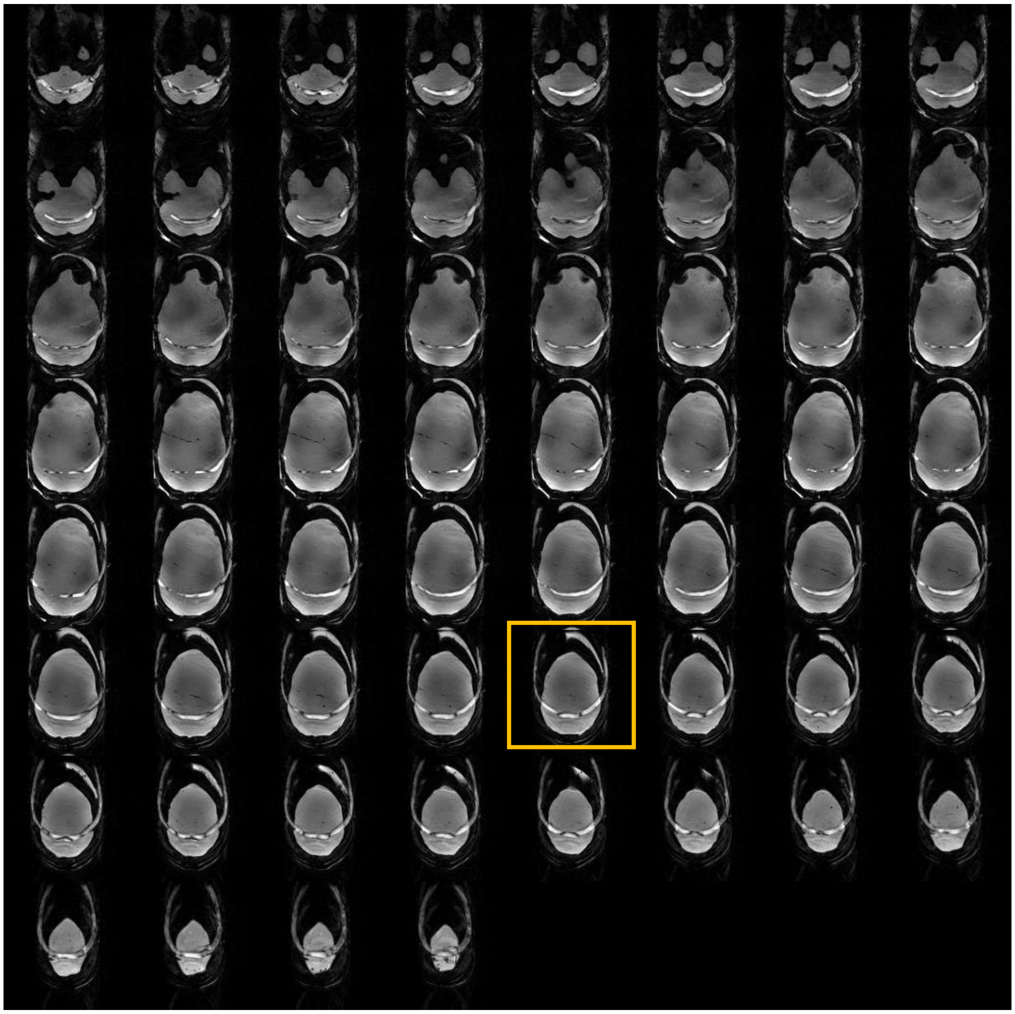

Figure S2: All (60) slices acquired without Fat Suppression. The orange overlay marks the slice shown in Figure S1.

S3. Example results from masked and non-masked lipid-sensitivity maps

In the current implementation we chose to generate the lipids sensitivity maps from an additionally acquired lipid-only scan (GRE scan with water suppression ON). Due to the lipids' localization their resulting sensitivity map automatically and conveniently included a spatial mask, although not a tight one (generated via an internal thresholding in the BART command we used). The lipids sensitivity map can also be generated from the same water image used for the water sensitivity map but shifted by the expected lipid shift. The focus of the current work is the reconstruction of the water image while removing the lipid signal without the need for a fat suppression pulse. Examining the water image, no significant differences were observed due to the different choice of the lipids' sensitivity map (see Figure S3a and S3b). For applications where the lipid image is of interest, further optimization of the sensitivity maps is required. Figure S3 shows an example slice also shown in Figure 4, here reconstructed with both options. The lipid signal is clearly observed in both cases (Figure S3a and Figure S3b), which also shows a good correlation with the GRE lipid signal (Figure S3c). However, since the lipid signal is low, any residual water signal in the lipids image has higher impact on the "lipids", thus requiring a better optimized reconstruction.

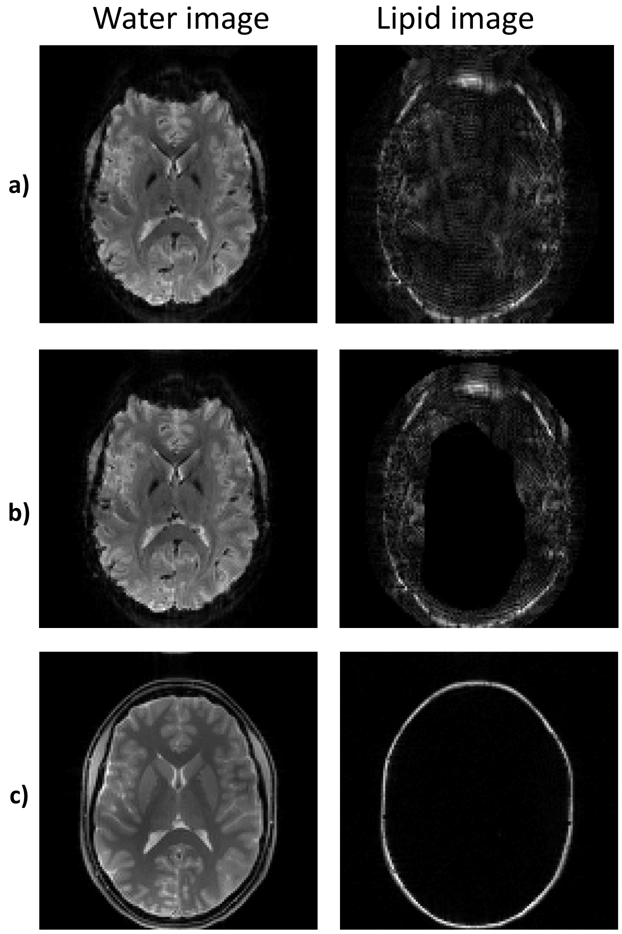

Figure S3: Water and lipid images reconstructed with masked and non-masked lipid sensitivity map. a) non-masked lipid sensitivity map, generated based on water GRE image shifted by the expected lipid signal shift, b) masked lipid sensitivity map, generated based on the lipid GRE image, c) water and lipid GRE images

S4. Analysis of the lipid artifact – simulation study

A simulation based on GRE reference scans – once with fat suppression (water reference image), and once with water suppression (lipid reference image) – was performed. In this

simulation the lipid image was shifted to match the EPI shift by Eq.2 (similarly to the shift performed in Figure 2). The GRE data and the respective sensitivity maps were used to reconstruct once the combined image with lipid artifact (simulating the "No Fat Suppression" image) and once the water image using the fat separation formulation discussed in this study. To examine the effect of the local phase at the true lipid position on the artifact strength, the simulation was repeated for a varied (global) phase added to the lipid image. The added phase simulates the local phase that can arise in a specific complex signal, for example due to different B_0 distributions, or from the lipid off-resonance itself. In order to better mimic the lipid signal contribution, the GRE short TE image was scaled by a factor accounting for the different T_2^* decay of the lipid compared to that of gray matter at the TE of EPI: ($e^{-\frac{TE_{EPI}}{T_{2\,lip}^{*}}} \Big/ e^{-\frac{TE_{EPI}}{T_{2\,GM}^{*}}}$). The T_2^*, from a separate volunteer multi-echo GRE scan, were estimated as 12.5±2.5 ms and 24.8±1.4 ms for the lipid layer and the gray matter, respectively. The deviation of the resulting image including the lipid artifact from the original water image was calculated. Figure S4 shows the comparison. It can be seen that due to the complex signal of the water and lipid and the specific sensitivity maps, the lipid artifact can result in either positive or negative extra signal. Figure S4.b shows the deviation at a representing point of the lipid artifact, variying between -23% to +23%. In addition, it can be seen that the artifact strength depends on the spatial location in the image (see the deviation map in Figure S4.c). The root-mean-square error (rmse) calculated for the water image extracted by the fat separation formalism is 1.7 and 1.4% in the two maximal error examples.

We also examined the effect of a small movement that may occur during the functional MRI experiment. We simulated a one pixel up movement relative to the sensitivity maps. The results are shown in FigS4.e with a one pixel shift relative to FigS4.c (and the same global phase). It can be seen that the deviation, without fat suppression now reaches 32%. The rmse of the water image extracted by the algorithm increased as well, however, reached only 3.3%.

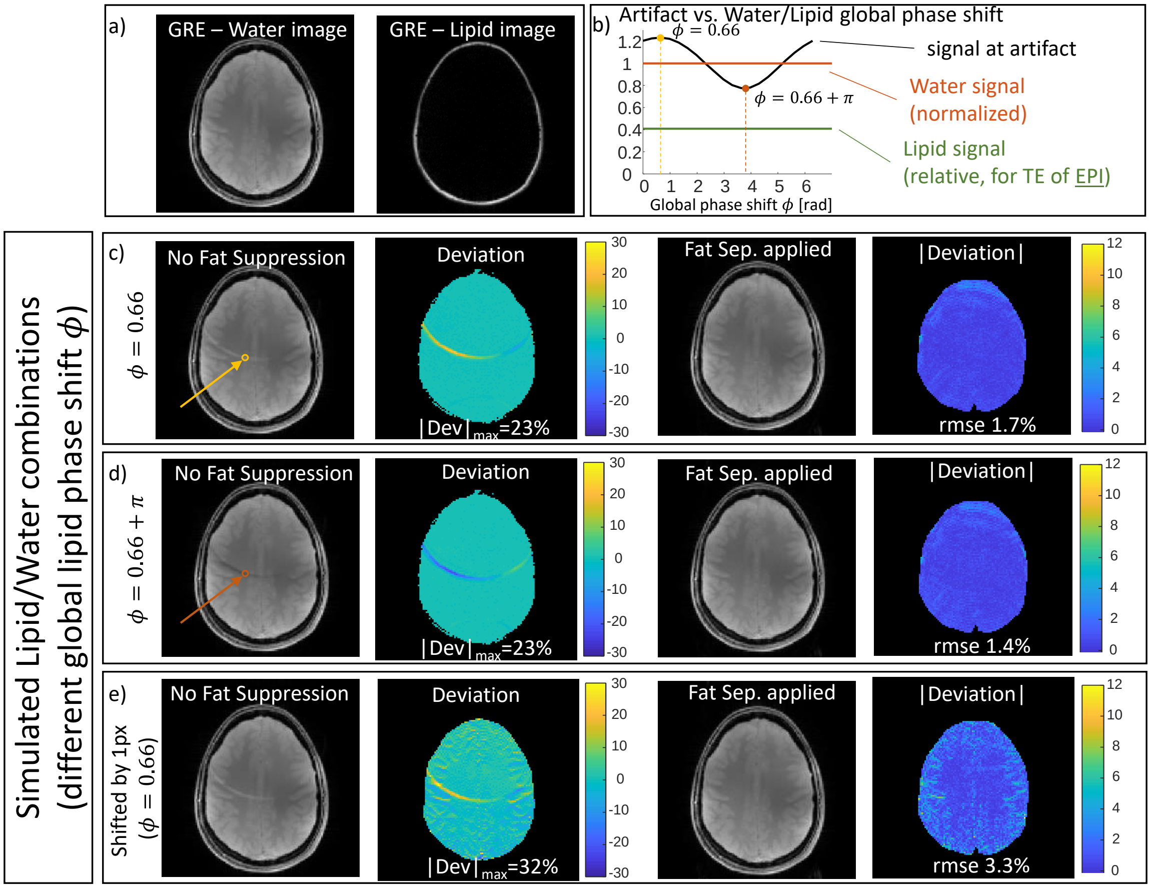

Figure S4: **Simulated Lipid/Water combined signal and resulting images.** a) GRE water and lipid reference images. b) Artifact at representing point (shown in (c) and (d)) versus added phase to the lipid image. c) Simulated results for ϕ=0.66 rad (maximal deviation), d) simulated results for ϕ=0.66+π rad (negative maximal deviation), e) simulated results for ϕ=0.66 rad plus simulated movement by one pixel. The images from left to right show – image if no Fat Suppression was applied, it's deviation map compared to the water reference image, the water image reconstructed by the fat-water separation formalism, and it's absolute deviation map from the water reference image.

S5. fMRI motor-task and resting-state experiments – comparison with and without Fat Suppression

Three volunteers were scanned to perform motor-task and resting-state fMRI. The experiments were repeated to include total six sets that included scans with and without Fat Suppression, for both the motor-task and the resting state. One of the sets in the motor-task was excluded due to severe movement during the scan.

The motor-task comprised of a finger tapping in right and left hands. The experiment included 8 right hand and 8 left hand blocks of finger tapping with total scan time of 5 minutes. The block durations were randomized, resulting in 17±3.5 sec block durations.

The experiment was repeated with fat-suppression and without it (but with fat-separation reconstruction). Figure S5 shows the three central slices in the motor activated area in a representing volunteer dataset with the t-test overlays for each hand. In addition, the figure compares the number of voxels found above different t-test thresholds (in absolute value), for all slices and for the three shown slices. The ratio of the number of voxels with Fat Separation compared to that with Fat Suppression is 1.43 for t-test of 2.4 (p-value of 0.99 confidence). The ratio further increases with the t-test threshold, however, for too small number of voxels the estimation is less reliable. We also calculated the tSNR for this experiment (Figure S6), which similarly to the shown in Fig.6 shows an increased tSNR in the Fat Separation case. The average increase in tSNR in the region of interest for motor fMRI (orange overlay in Fig. S6) is 1.6±0.7.

In addition, relative signal change (ΔS) was estimated for the same number of voxels in each experiment. In order to choose voxels with high correlation to the stimuli, voxels from all slices with a t-test of 5 and higher for the case with Fat Suppression were selected. The same number of voxels, with the highest t-test values, were also chosen in the Fat Separation case. The average ΔS for the above voxels as a function of the scan time and summed for all stimuli is shown in Fig. S7. The maximal ΔS was calculated by averaging over 5 time points around the maximal point. The standard error (StdErr) was calculated as the average of the standard error over the same 5 time points. We defined contrast to noise ratio (CNR) as ΔS/StdErr. Table S1 summarizes the averaged maximal ΔS, the standard error and the CNR calculated for the repeated scans in three volunteers.

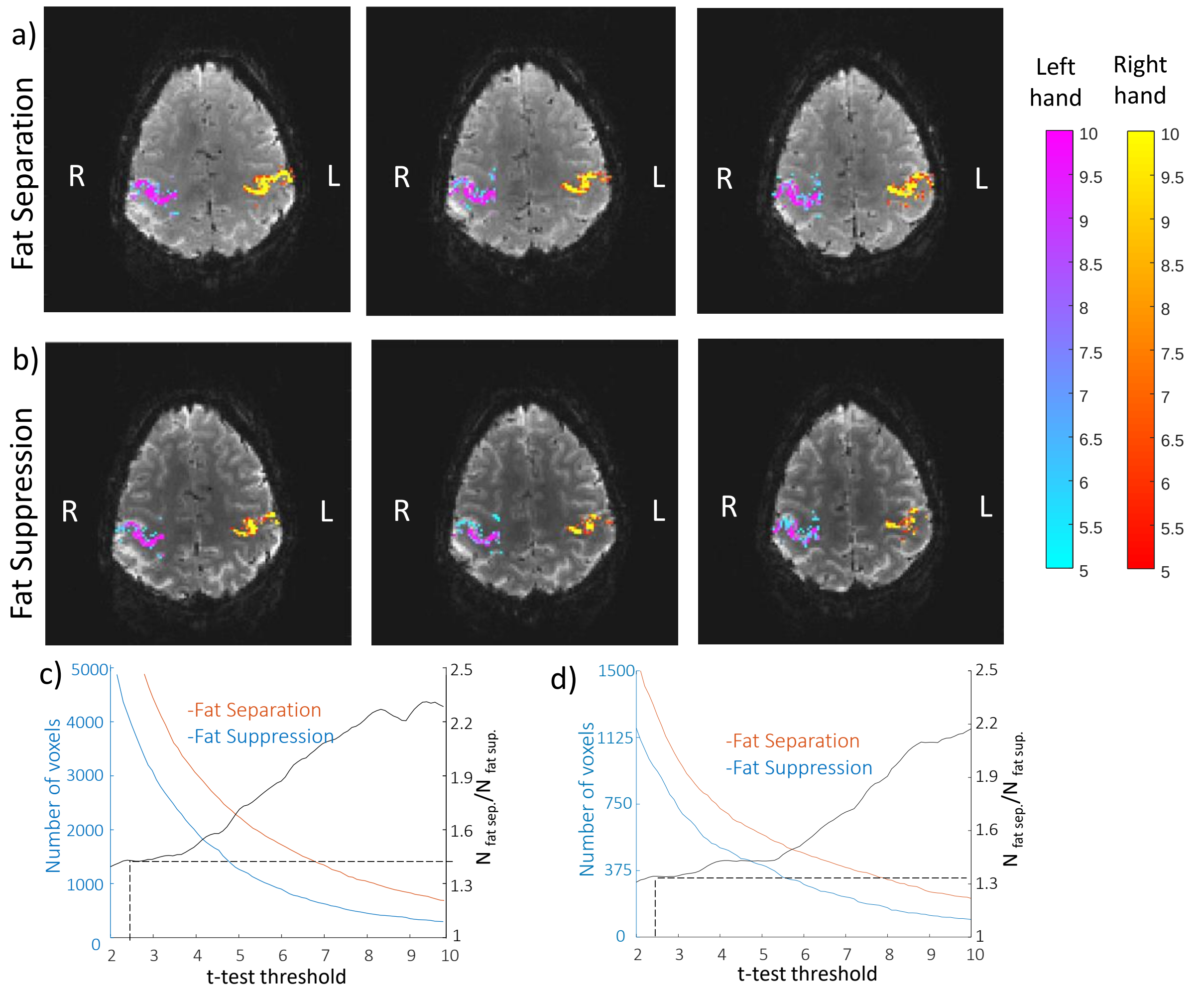

Figure S5: **fMRI motor-task experiment – comparison of the t-test overlay in Fat Separation (a) and Fat Suppression (b) cases.** The t-test overlay is shown on top of the representing three slices in the motor area (the left-hand t-test with “cool” colormap and the right-hand with “autumn” colormap). At the bottom, a plot of the number of voxels above each t-test value is shown for each case (blue and red) – for all slices (c) and for the above three slices (d). On the same plot, the ratio between the number of voxels in Fat Separation / Fat Suppression cases is shown in black.

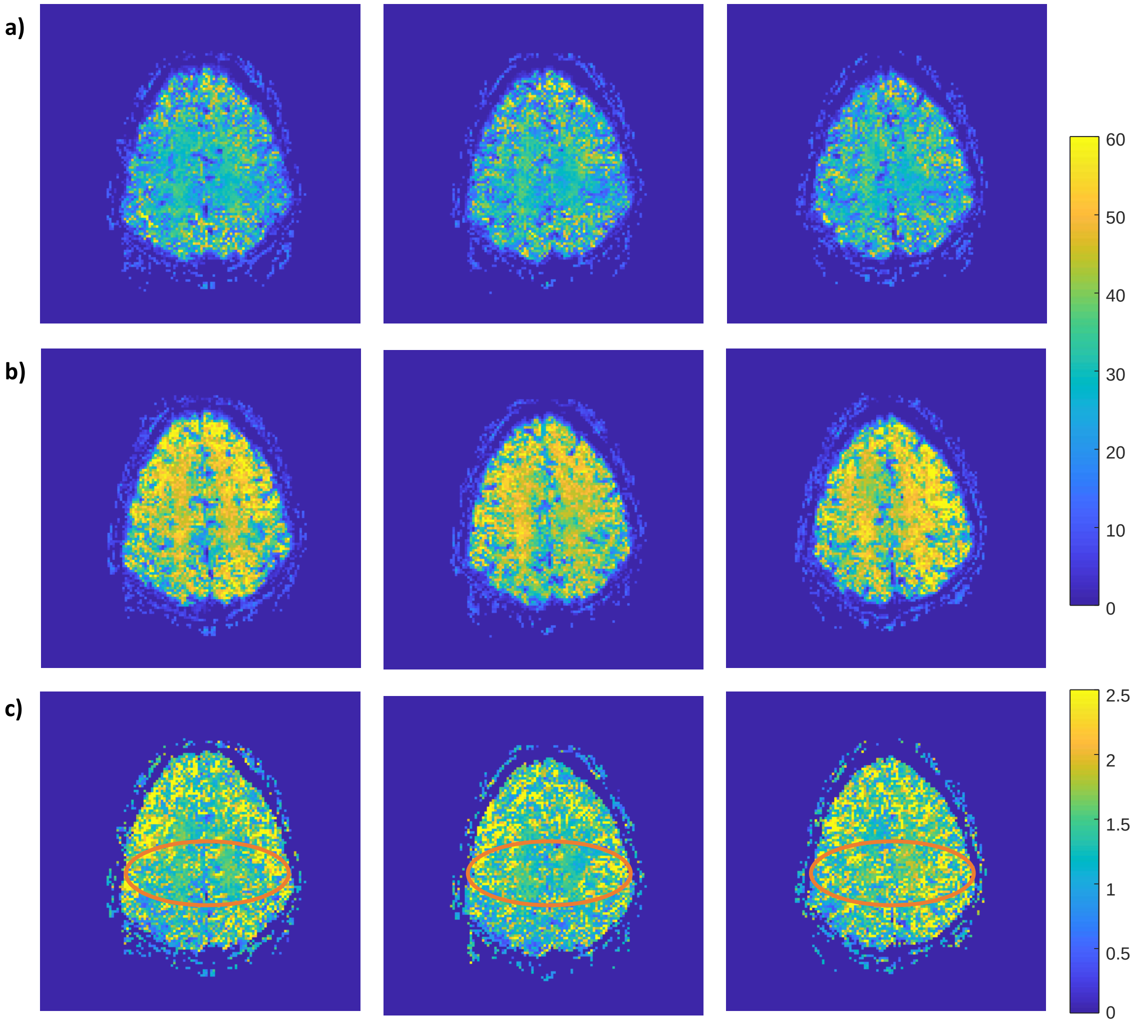

***Figure S6:* tSNR maps in the three slices shown in Fig.S5.** a) Fat Suppression case, b) Fat Separation case, c) Ratio of the Fat Separation / Fat Suppression. Orange overlay shows the region of interest that the average and standard deviation were calculated.

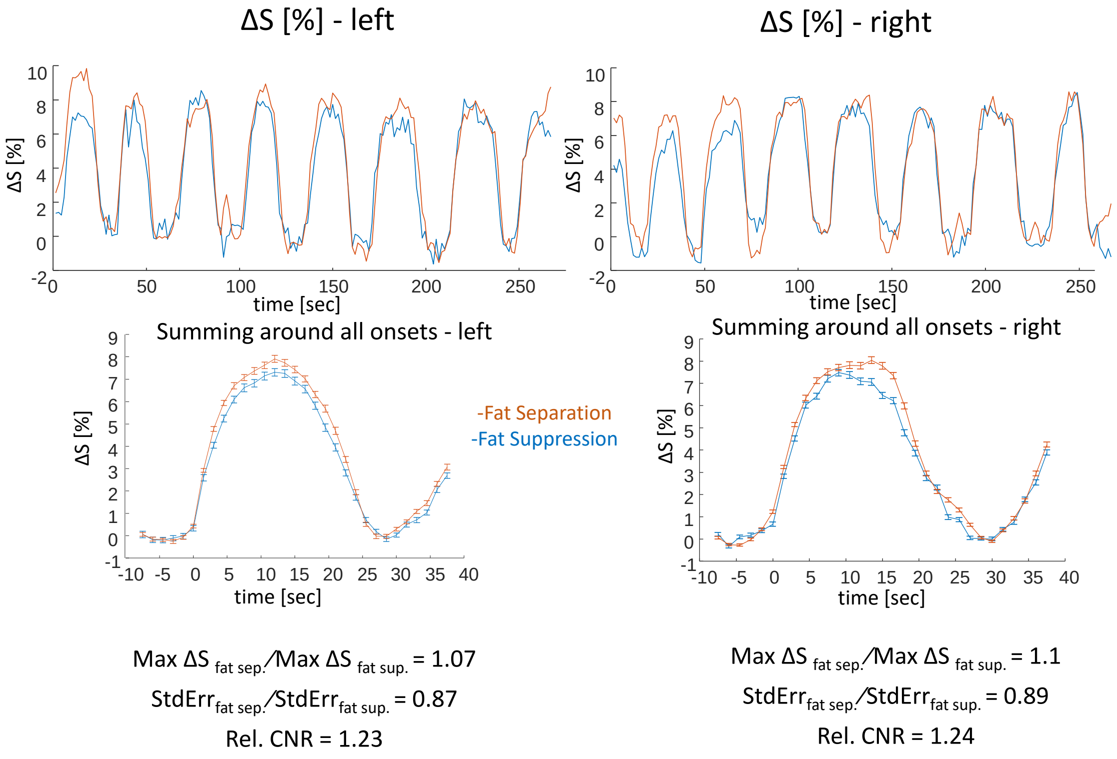

Figure S7: **Signal change (ΔS) as function of time.** Upper row shows the full experiment duration. In the bottom row the signal change is summed over all stimuli, where time zero is the onset of the stimuli. Maximal signal change is calculated for the bottom plots as an average over 5 pixels around the maximal value of each.

Table S1: Relative CNR changes (CNR=ΔS/StdErr) in voxels with t-test>5				
	Number of voxels (averaged for each volunteer, includes right and left sides, 7 slices in the motor activated area)	ΔS±StdErr [%] Fat Separation	ΔS±StdErr [%] Fat Suppression	Relative CNR change (Fat Sep. vs Fat Sup.)
Vol #1	926	5.758±0.087	6.100±0.124	1.34
Vol #2	770	6.195±0.077	6.204±0.106	1.48
Vol #3	958	5.395±0.064	5.375±0.065	1.02
All volunteers	885	5.783±0.074	5.893±0.0982	1.30

The resting-state experiment included 5 minutes scans (repeated 6x2 times). The analysis was performed with SPM, including motion correction and smoothing of FWHM=4 mm in all directions. A region in the Posterior Cingulate Cortex (PCC) was selected for seed-based functional connectivity analysis, as representing region of the Default Mode Network (DMN). Figure S8 show representing t-test overlay of the PCC seed-based connectivity analysis. Figure S9 shows tSNR maps for all three volunteers. Table S2

summarizes averaged ratio of the tSNR with Fat Separation and Fat Suppression in the PCC region as shown in Figure S9 by the black overlay. The average ratio (from Table S2) over all volunteers is 1.27±0.29.

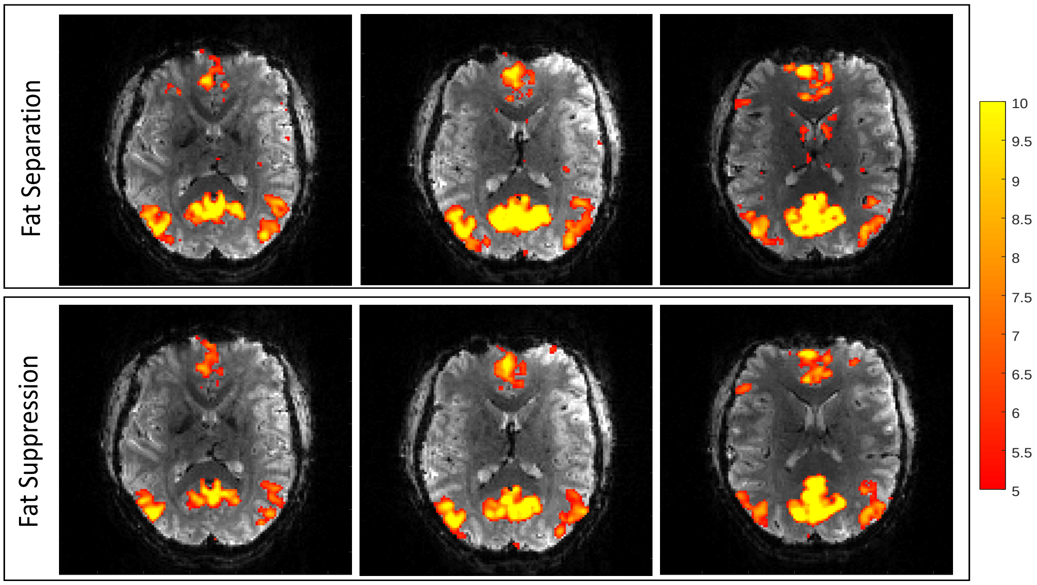

Figure S8: **Seed-based connectivity for PCC area with Fat Separation and Fat Suppression.** Three slices in the PCC region are shown.

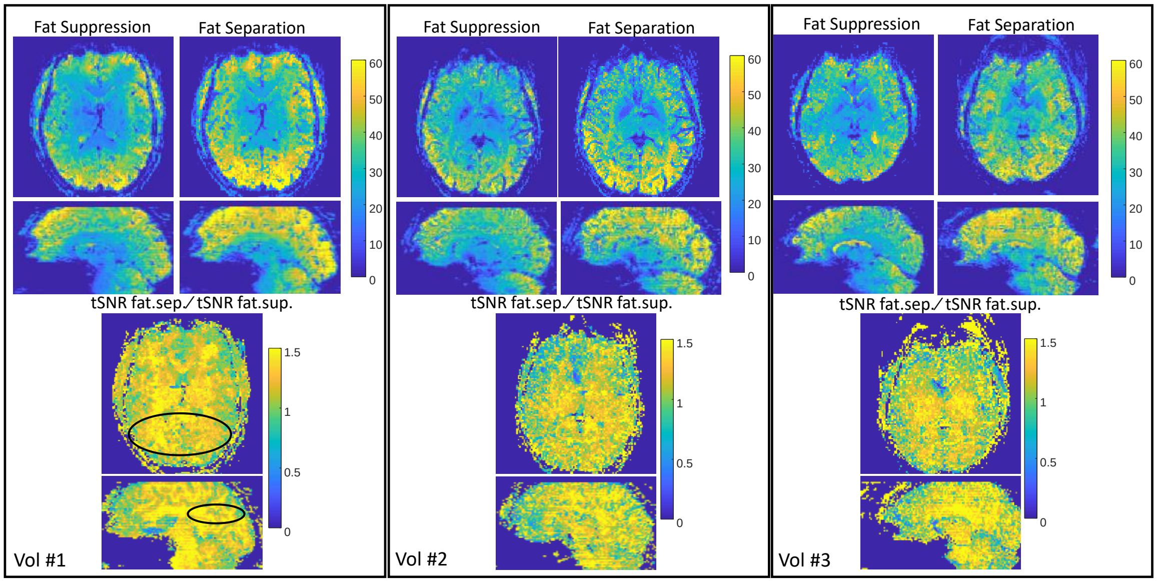

Figure S9: **tSNR in resting-state fMRI**. Average maps for three volunteers. Transversal and sagittal main cross sections are shown. The upper two rows show the tSNR maps and the bottom two rows the ratio of the tSNR Fat.Sep./tSNR Fat.Sup.

Table S2: tSNR ratio (tSNR Fat.Sep./tSNR Fat.Sup) in resting state (average in the PCC area – shown in Fig.S9)	
Vol #1	1.25±0.27
Vol #2	1.29±0.24
Vol #3	1.27±0.37
All volunteers	1.27±0.29
